# Genomic analysis of 1,25-dihydroxyvitamin D_3_ action in mouse intestine reveals compartment and segment-specific gene regulatory effects

**DOI:** 10.1016/j.jbc.2022.102213

**Published:** 2022-06-30

**Authors:** Rohit Aita, Dennis Aldea, Sohaib Hassan, Joseph Hur, Oscar Pellon-Cardenas, Evan Cohen, Lei Chen, Noah Shroyer, Sylvia Christakos, Michael P. Verzi, James C. Fleet

**Affiliations:** 1 Department of Genetics, Human Genetics Institute of New Jersey, Rutgers Cancer Institute of New Jersey, Institute of Food, Nutrition, and Health, EOHSI, Rutgers University, New Jersey, USA; 2 Department of Medicine, Section of Gastroenterology and Hepatology, Baylor College of Medicine, Houston, Texas, USA; 3 Department of Microbiology, Biochemistry, and Molecular Genetics, Rutgers, The State University of New Jersey, New Jersey Medical School, Newark, New Jersey, USA; 4 Department of Nutritional Science, University of Texas, Austin, Texas, USA

**Keywords:** vitamin D, vitamin D receptor, steroid hormone receptor, transcription, transcription factor, transcription enhancer, intestinal epithelium, colon, small intestine, genomics, 1,25(OH)2D3, 1,25-dihydroxyvitamin D3, CAR, constitutive androstane receptor, DEG, differentially expressed gene, FDR, false detection rate, PGE2, prostaglandin E2, PXR, pregnane X receptor, SI, small intestine, VD, 1,25-dihydroxyvitamin D, VDR, vitamin D receptor

## Abstract

1,25-dihydroxyvitamin D (VD) regulates intestinal calcium absorption in the small intestine (SI) and also reduces risk of colonic inflammation and cancer. However, the intestine compartment-specific target genes of VD signaling are unknown. Here, we examined VD action across three functional compartments of the intestine using RNA-seq to measure VD-induced changes in gene expression and Chromatin Immunoprecipitation with next generation sequencing to measure vitamin D receptor (VDR) genomic binding. We found that VD regulated the expression of 55 shared transcripts in the SI crypt, SI villi, and in the colon, including *Cyp24a1*, *S100g*, *Trpv6*, and *Slc30a10*. Other VD-regulated transcripts were unique to the SI crypt (162 up, 210 down), villi (199 up, 63 down), or colon (102 up, 28 down), but this did not correlate with mRNA levels of the VDR. Furthermore, bioinformatic analysis identified unique VD-regulated biological functions in each compartment. VDR-binding sites were found in 70% of upregulated genes from the colon and SI villi but were less common in upregulated genes from the SI crypt and among downregulated genes, suggesting some transcript-level VD effects are likely indirect. Consistent with this, we show that VD regulated the expression of other transcription factors and their downstream targets. Finally, we demonstrate that compartment-specific VD-mediated gene expression was associated with compartment-specific VDR-binding sites (<30% of targets) and enrichment of intestinal transcription factor–binding motifs within VDR-binding peaks. Taken together, our data reveal unique spatial patterns of VD action in the intestine and suggest novel mechanisms that could account for compartment-specific functions of this hormone.

Vitamin D is an important nutrient with critical regulatory actions on intestinal physiology and function (1). Vitamin D is metabolized to become 1,25-dihydroxyvitamin D_3_ (1,25(OH)_2_D_3_), a hormone that activates the vitamin D receptor (VDR) to mediate the transcription of target genes. VDR-mediated gene transcription is a multistep process that involves VDR binding to target genes at both active promoters and distal regulatory elements as well as recruitment of coregulatory proteins (2). Although 1,25(OH)_2_D_3_ has been reported to regulate physiological processes in many tissues, the highest levels of VDR are present in the intestine, the major 1,25(OH)_2_D_3_ target tissue (3, 4). Studies in VDR null mice showed that deletion of VDR causes the loss of active calcium absorption in the proximal intestine, leading to hypocalcemia and rickets (5). In addition, intestine-specific transgenic expression of VDR in VDR null mice normalized calcium absorption, serum calcium, and prevented the development of rickets (6, 7). These findings indicate that a primary role of VDR and 1,25(OH)_2_D_3_ signaling during growth is the regulation of intestinal calcium absorption needed for calcium homeostasis and bone mineralization. Although most studies have focused on the duodenum, our recent studies have shown that the distal segments of the intestine also play an important role in VDR-mediated intestinal calcium absorption and bone mineralization (7, 8, 9).

In addition to maintenance of calcium homeostasis, many other beneficial intestinal effects of 1,25(OH)_2_D_3_ have been described including anti-inflammatory effects, maintenance of intestinal barrier function, and protection against colitis and colon cancer, suggesting the existence of multiple, diverse 1,25(OH)_2_D_3_ functions across the length of the intestine (1). In addition to functional differences that exist along the proximal-to-distal axis, the proximal segments of the intestine have epithelial cells organized along a crypt–villus axis. Several studies have shown that 1,25(OH)_2_D_3_ action varies along the crypt–villus axis. In duodenal mid-villus cells, 1,25(OH)_2_D_3_ rapidly stimulates calcium extrusion (10) but slower effects of 1,25(OH)_2_D_3_ on crypt cells program the intestine for improved calcium absorption as the cells differentiate and migrate into the villus (11). Meanwhile, VDR loss increases colon epithelial cell proliferation and alters the contribution of Lgr5+ stem cells to the maintenance of the intestinal epithelium (12, 13). In spite of the recognized regulatory role of 1,25(OH)_2_D_3_–VDR signaling in intestinal biology, the mechanisms involved in VDR-mediated regulation of these diverse functions remain incomplete and genomic studies of 1,25(OH)_2_D_3_ action in the intestine are sparse. In addition, the diversity and complexity of 1,25(OH)_2_D_3_ signaling with respect to proximal–distal and crypt–villus axes have not as yet been evaluated when considering intestinal 1,25(OH)_2_D_3_ action.

In this study, we used a series of complementary genomic tools (*i.e.*, RNA-seq, VDR ChIP-seq, ATAC-seq) to identify 1,25(OH)_2_D_3_-responsive target genes across the proximal-distal and small intestine (SI) crypt–villus axis. Our findings show that while a number of 1,25(OH)_2_D_3_-regulated genes are common across SI villus, SI crypt, and colon, the majority of 1,25(OH)_2_D_3_-regulated transcripts have compartment-restricted regulation patterns. Gene ontology (GO) and pathway analysis of the 1,25(OH)_2_D_3_-regulated transcripts from each compartment indicated regulation of unique biological functions, independent of calcium homeostasis, including regulation of RNA metabolic processes, tight junctions, metabolism of xenobiotics, lipid metabolic processes, and HIF1 signaling. However, not all VDR-regulated genes have VDR-binding peaks, suggesting that some transcript levels effects of 1,25(OH)_2_D_3_ are indirect and may be due in part to the contribution of other transcription factors. Our findings are the first to define 1,25(OH)_2_D_3_-molecular actions across the critical proximal–distal and crypt–villus axes that define the functional characteristics of the intestine and suggest novel mechanisms that may account for intestine compartment-specific functions of 1,25(OH)_2_D_3_.

## Results

We confirmed the quality of our isolation of the various intestinal segments in two ways. First, we visually examined the small intestinal villus and crypt preparations to confirm that they were pure (see Fig. S1 for representative pictures of the isolated small intestinal crypts and villi). In addition, we examined our RNA-seq data to identify the transcript level differences across the three compartments. As expected, there were many differentially expressed genes between the small intestinal villus [5,114, 1% false detection rate (FDR), 2-fold change] or crypts (3554 differentially expressed gene (DEG)) and the colon. This included 135-fold higher mRNA levels of the SI marker lactase (*Lct*) in the villus *versus* colon and a 189-fold greater expression of the colon marker, carbonic anhydrase 1 (*Car1*) mRNA, in colon *versus* villus (Table S1). In the 3669 DEG we observed between the crypt and villus compartments, we observed that transcripts for markers of differentiated small intestinal epithelial cells were enriched in the SI villus (*Lct*, 3.49 up, *S100g*, 3.56 up, *Trpv6*, 3.71 up), while expression of the intestinal stem cell marker *Lgr5* was elevated significantly in crypts (+12.2 fold). Collectively, these compartment-level differences in transcript levels confirm the quality of our isolation procedure.

**Figure 1:**
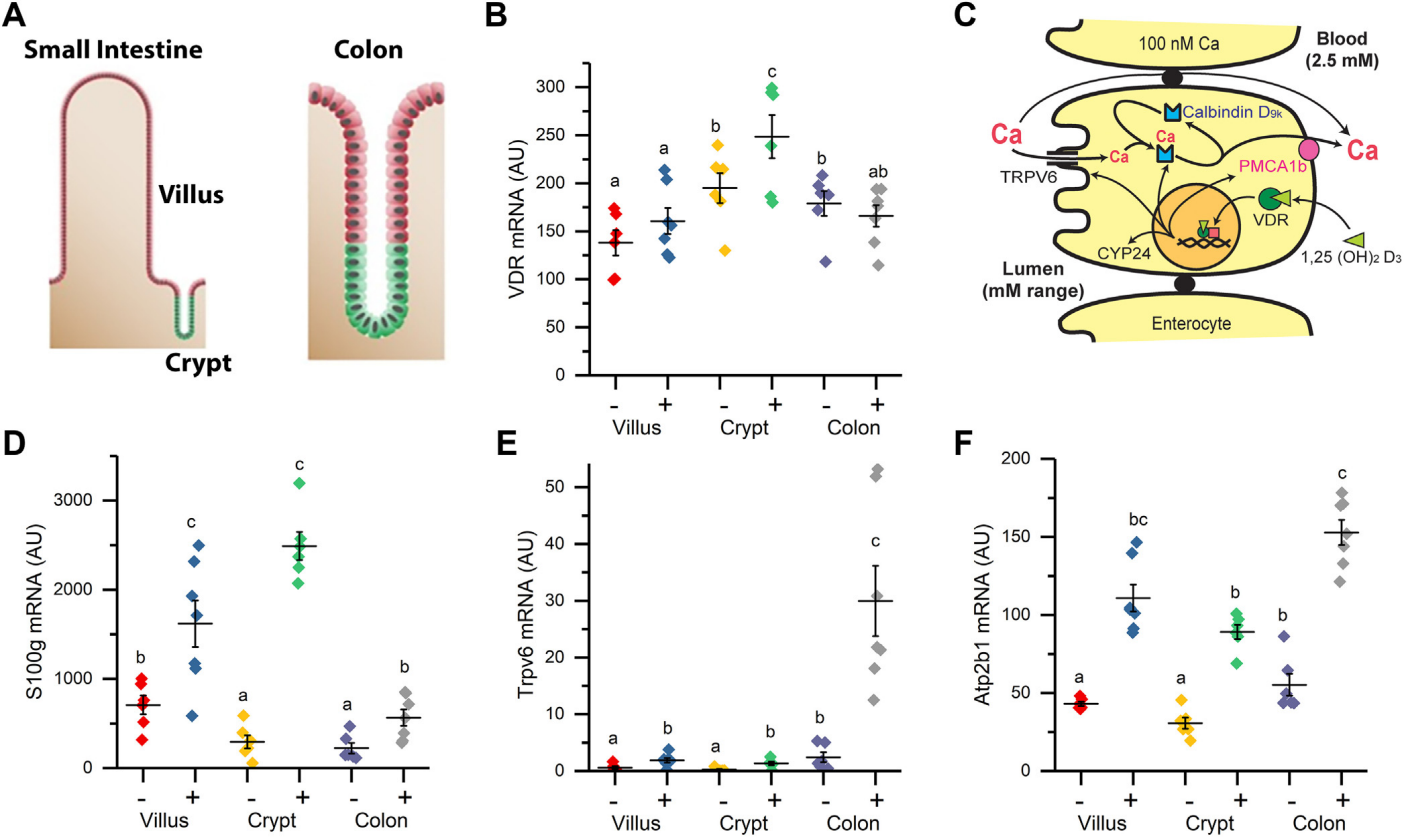
**RNA-seq analysis identified 1,25(OH)**_**2**_**D**_**3**_**-induced classical 1,25(OH)**_**2**_**D**_**3**_**targets that mediate intestinal Ca absorption.***A*, schematic depicts intestinal compartments investigated for RNA-seq analysis along the crypt/villus and proximal/distal axis; *B*, basal and 1,25(OH)_2_D_3_ -mediated regulation of *Vdr* mRNA in the three intestinal compartments. *C*, a model for 1,25(OH)_2_D_3_-regulated transcellular intestinal calcium absorption where TRPV6 permits Ca entry, Calbindin D_9k_ (coded by *S100g*) buffers intracellular Ca, and PMCA1b (encoded by *Atp2b1)* facilitates ATP-dependent Ca extrusion from the cell. *C-F*, mRNA levels for *Trpv6, S100g, and Atp2b1* as regulated by 1,25(OH)_2_D_3_ treatment (10 ng/g, 4 h) in the three intestinal compartments. In panels (B), (D), (E), and (F), data groups with different letter superscripts are significantly different from one another (*p* < 0.05, ANOVA followed by Tukey’s HSD). 1,25(OH)2D3, 1,25-dihydroxyvitamin D3.

**Figure 2:**
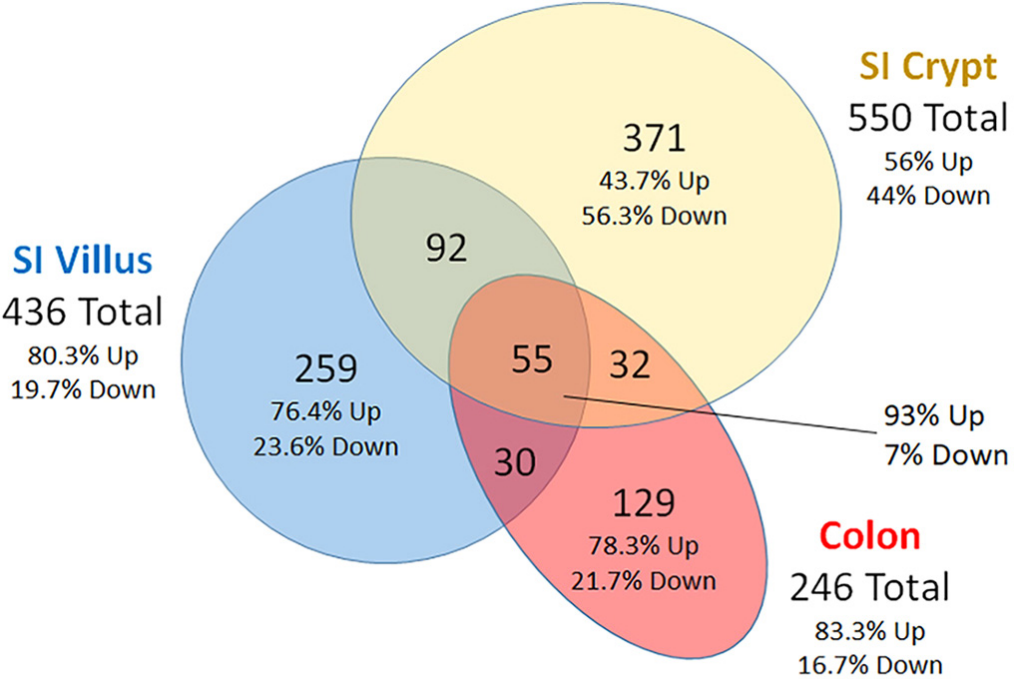
**Venn diagram depicting significantly differentially expressed transcripts in the small intestine crypt, small intestine villus, or colon.** Mice were treated with 1,25(OH)_2_D_3_ (10 ng/g, 4 h) and RNA from the three compartments was used for RNA-seq analysis. Differential expression was determined using DeSeq2 (5% FDR). The percentage of upregulated (UP) and downregulated transcripts (DN) are prevented for the total number of differentially expressed transcripts by tissue and for the compartment-specific transcripts. 1,25(OH)2D3, 1,25-dihydroxyvitamin D3.

A summary of the impact of 1,25(OH)_2_ D treatment on intestinal gene expression is presented in Table S2. As expected, 1,25(OH)_2_D_3_ treatment significantly induced the expression of several genes known to be involved in intestinal calcium absorption; *Trpv6*, *S100g*, and *Atp2b1* (Fig. 1). In addition, our analysis showed that *Vdr* mRNA levels were not dramatically different across segments nor were they strongly regulated by 1,25(OH)_2_ D treatment (Fig. 1*B*). Figure 2 shows that 968 transcripts were differentially regulated by 1,25(OH)_2_ D across the three compartments at the 5% FDR (A Venn Diagram showing the differentially expressed genes at 10% FDR is provided as Fig. S2.) Only 55 of these genes were common across all compartments (including the known 1,25(OH)_2_D_3_ target genes *Cyp24a1*, *Trpv6, S100g, Slc30a10*, and *Atp2b1*), while 78% of the 1,25(OH)_2_D_3_-regulated transcripts were specific to just one compartment. Ninety three percent of the 55 common targets were upregulated by 1,25(OH)_2_D_3_ treatment. Similarly, more than 80% of 1,25(OH)_2_D_3_-regulated genes in the SI villus and colon were induced. In contrast, only 56% of SI crypt transcripts were upregulated.

We compared our 1,25(OH)_2_D_3_-differentially expressed gene list with data on 1,25(OH)_2_D_3_-regulated transcripts in the SI that was previously published by Lee *et al*. (14). Of the 719 DEG reported by them, 486 transcripts were identified in our intestinal RNA-seq data. One hundred seventy-nine of these matched to the 10% DEG list from at least one of the intestinal compartments (Table S2 and Fig. S3). Thirty eight transcripts were upregulated by 1,25(OH)_2_D_3_ in all three compartments and also in the Lee *et al*. dataset, including *S100g, Trpv6, Atp2b1, Cyp24a1,* and *Slc30a10*.

We examined the 1,25(OH)_2_D_3_-regulated gene list from each compartment for enrichment of GO terms and pathways (Table 1 and Table S3 (Pathways), S4 (GO Up enrichment), and S5 (GO down enrichment)). Distinct functional categories of genes were identified for each compartment, including enrichment of GO terms for “lipid metabolic processes” and “ion transport” in villus, terms related to rRNA, RNA, and ncRNA processing in the crypts, and “Negative regulation of cell population proliferation” and “Regulation of Cell migration” in colon.

**Table 1:** Summary of GO enrichment for 1,25(OH)_2_D_3_-regulated genes by compartment

Tissue	Enrichment	Topic	Genes in DEG list
Crypt	Up	rRNA/RNA/ncRNA processing	76
		Ribosome/cell component biogenesis	103
Crypt	Down	Response to chemical stimulus	140
		Positive regulation of ion transport	54
Villus	Up	Ion transport	103
		Lipid metabolic processes	72
Villus	Down	Primary metabolic processes	78
		Cell activation	22
Colon	Up	Negative regulation of cell population proliferation	39
		Regulation of cell migration	43
Colon	Down	Cell developmental processes	33
		Regulation of cellular component organization	24

**Figure 3:**
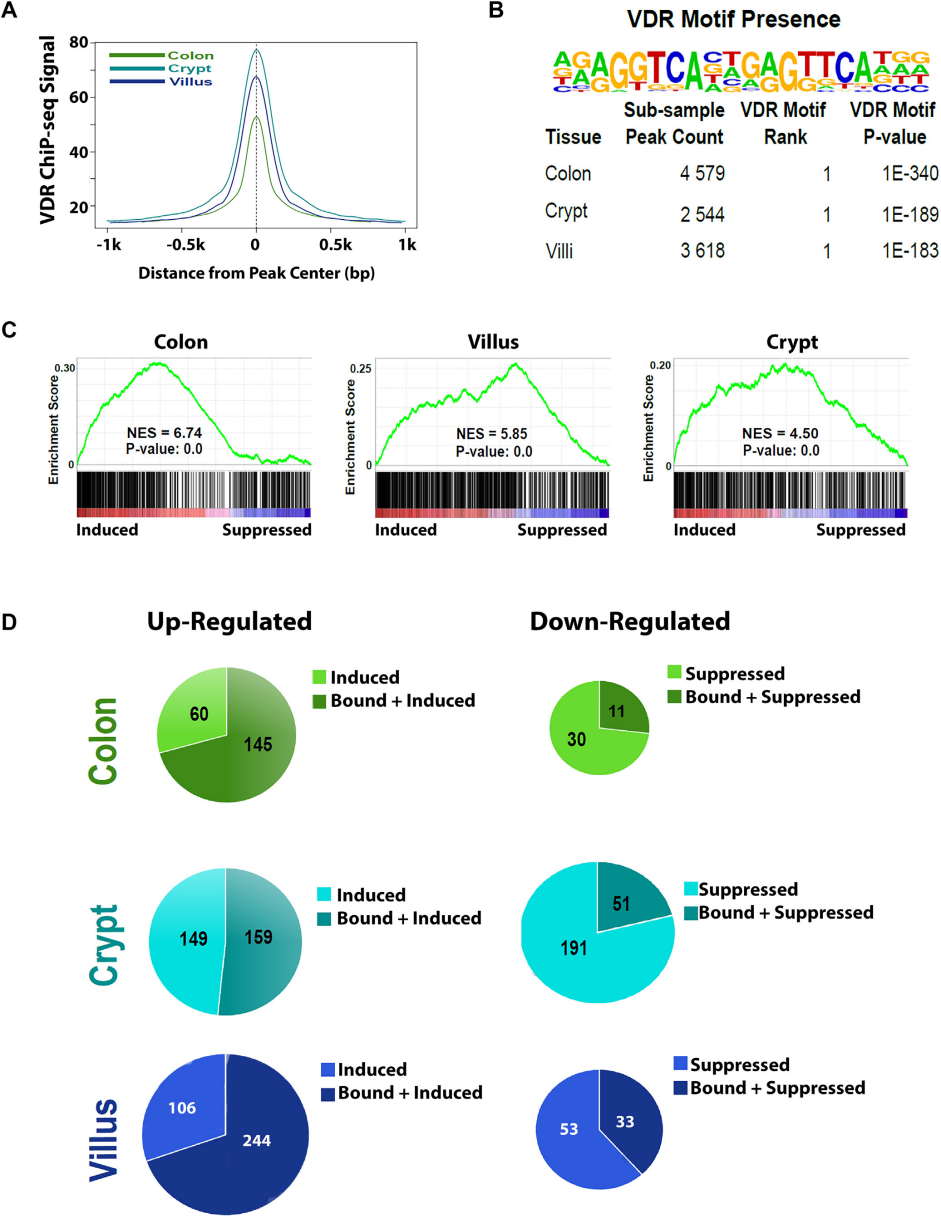
**ChIP-seq analysis of VDR binding and its relation to differentially expressed genes in each compartment.** Mice were treated with 1,25(OH)_2_D_3_ (10 ng/g body weight) and tissues were collected for VDR ChIP-seq after 1 h. *A*, composite ChIP-seq signal at regions identified as binding VDR in the three intestinal compartments show strong and centered sequence reads. *B*, HOMER motif analysis identified transcription factor–binding motifs enriched at VDR-binding regions from each tissue. Twenty percent of the ChIP peaks identified in each tissue were randomly sampled for motif analysis. The canonical VDR motif was the most prevalent in each tissue. *C*, RNA-seq analysis was conducted in each tissue and genes linked to colon, crypt, or villus binding (within 10 kb) were correlated utilizing GSEA analysis. GSEA plots indicate VDR-binding sites are nearby genes that are 1,25(OH)_2_D_3_-induced in each tissue. *D*, pie charts depict genes upregulated (Left) or downregulated (Right) by 1,25(OH)_2_D_3_ treatment (5% FDR) and the subset of these bound by VDR in the colon, small intestinal crypt, or small intestinal villus. 1,25(OH)2D3, 1,25-dihydroxyvitamin D3; VDR, vitamin D receptor.

Using VDR ChIP-seq, we found many 1,25(OH)_2_D_3_-induced VDR-binding peaks in each intestinal compartment: 12,719 in SI crypt, 18,083 in SI villus, and 22,888 in colon. The ChIP-Seq signal was similar across compartments and VDR ChIP peaks averaged ∼ 1000 bp wide (Fig. 3*A*). Included in our VDR ChIP-seq peaks were the previously reported VDR-binding peaks in the *Cyp24a1* gene (TSS at -0.2 kb and downstream enhancer peaks at +35, +37, +39, and +43 kb); the *Trpv6* gene (at -2, -4 kb); and *Slc30a10* gene (*e.g.* robust peaks at +29, +32, and +48 kb) (14). More than 60% of the VDR-binding peaks in the SI villus and crypt and 44% of the VDR-binding peaks in the colon coincided with the ATAC-Seq peaks from these same tissues (See Fig. S4). In contrast, some 1,25(OH)_2_D_3_-induced VDR peaks did not coincide with an ATAC-seq peak and this suggests that 1,25(OH)_2_D_3_ treatment revealed regulatory sites that were either silent under basal conditions or under the ATAC detection limit in untreated mouse intestine (*e.g.* Fig. S5 for the *Slc30a10* gene).

An evaluation of the VDR-binding peaks for known transcription factor binding site motifs revealed that the VDR-RXR DR3 motif was the most enriched motif in all three compartments (Fig. 3*B* and Table S6)). In addition, 16 other motifs for intestine-expressed transcription factors were enriched in the VDR ChIP-seq peaks (5% FDR, >1.5 fold enrichment over background DNA), including motifs for RAR, Bach1, HNF4a, JUN, and FOSL2 (in peaks from all three compartments); GATA4, YY1, MAFK (crypt and villus); HNF1, CEBP (crypt), and CDX2, TCF3, THRa (colon) (Fig. S6).

We attributed the VDR-ChIP peaks to their nearest neighbor gene using GREAT in GSEA. This data was then used to identify the genes differentially expressed by 1,25(OH)_2_D_3_ that also had a VDR-binding peak associated with them (Fig. 3*D*). Approximately, 70% of the 1,25(OH)_2_D_3_-induced transcripts in SI villus and colon had a VDR-binding peak. In contrast, only 52% of 1,25(OH)_2_D_3_-induced crypt transcripts had VDR-binding peaks, while even fewer 1,25(OH)_2_D_3_-suppressed transcripts had them (villus 38.4%; colon 26.8%; SI crypt 21.1%). This suggests that the regulation of many genes, especially those induced in the crypt and suppressed in all compartments, were not direct 1,25(OH)_2_D_3_ target genes but may be a consequence of an upstream 1,25(OH)_2_D_3_-regulated event.

**Figure 4:**
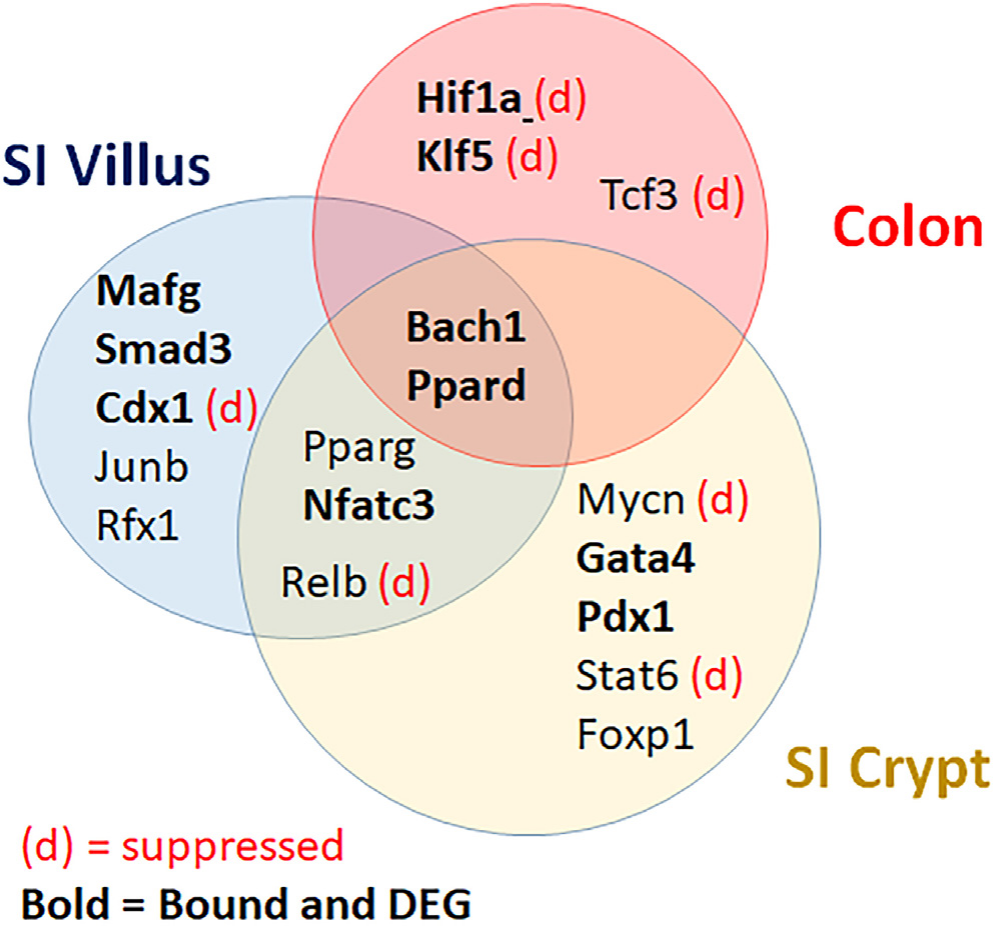
**1,25(OH)**_**2**_**D**_**3**_**-regulated transcripts for intestinal transcription factors and their relationship to the three intestinal compartments examined**. RNA-seq identified 1,25(OH)_2_D_3_ regulated transcription factor mRNA. Downregulated transcripts are identified with a *red* (d). Transcription factors whose downstream target genes were enriched in the list of 1,25(OH)_2_D_3_-regulated transcripts are identified with *bold* text. 1,25(OH)2D3, 1,25-dihydroxyvitamin D3.

One possibility for how 1,25(OH)_2_ D treatment could alter transcript levels independent of VDR binding to a gene regulatory region is that 1,25(OH)_2_D_3_ regulates the expression of other transcription factors that have 1,25(OH)_2_D_3_-independent downstream actions. Consistent with this hypothesis, bioinformatic analysis identified the protein class “transcription factors” as enriched in the 1,25(OH)_2_D_3_-regulated genes from crypt and colon (Table S7). In addition, we found that, of the transcription factors expressed in the colon (n = 307) or the SI (n = 348) of mice (15), 50 were differentially expressed by 1,25(OH)_2_D_3_ treatment (16 induced and six suppressed transcription factor genes had a VDR binding site). Of the 50 1,25(OH)_2_D_3_-regulated transcription factor messages, 18 had enrichment of their downstream target genes in our dataset (Fig. 4 and Table S8).

**Figure 5:**
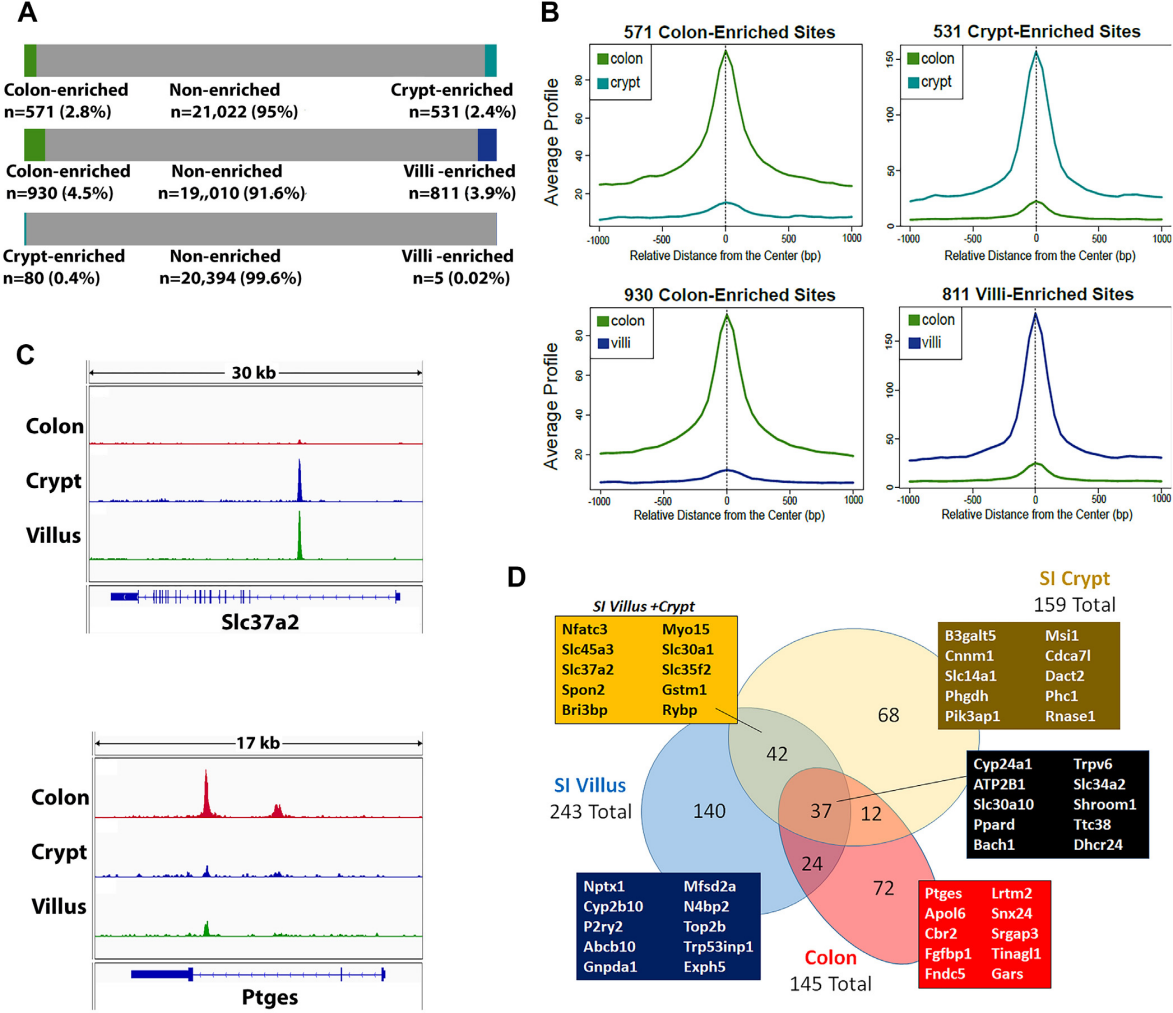
**VDR binds similar genomic regions in the duodenal crypts and villus but differential VDR binding is seen in proximal *versus* distal gut**. *A*, diffbind analysis was performed using VDR ChIP-seq replicates (n = 4) measuring VDR binding to the small intestine crypt, small intestine villus, or colon epithelium. Peaks with differential binding between compartments were identified (5% FDR). *B*, composite VDR ChIP-seq signal comparing the peaks with differential VDR binding in comparisons between colon and small intestine crypt or villus peaks. *C*, examples of genes with compartment-specific differential Vdr binding; *Slc37a2* has VDR peaks in small intestine but not colon; *Ptges* has robust peaks in colon but not small intestine. *D*, venn diagram of genes that are 1,25(OH)_2_D_3_-induced and have VDR-binding sites in the three compartments. Boxes show 10 representative genes for various compartments or overlap groups. 1,25(OH)2D3, 1,25-dihydroxyvitamin D3; VDR, vitamin D receptor.

We next used Diffbind to compare VDR peaks across the three compartments to test whether compartment-specific regulation of genes by 1,25(OH)_2_ D treatment was due to differential binding of VDR to specific regulatory sites. Differential VDR binding was minimal between the small intestinal crypt and villus (80 crypt-enriched peaks, five villus-enriched peaks). In contrast, there were several hundred VDR peaks that were differentially enriched in either the colon or SI (Fig. 5*A* with images of the enriched peak profile in Fig. 5*B*). As shown in Figure 3, VDR peaks are more common for the induced genes so we evaluated the number of DEG with VDR-binding peaks that had both compartment-specific binding and induced expression. Our data show that fewer than 30% of the compartment-specific, differentially regulated transcripts also had compartment-specific differential VDR binding. This includes genes like *Slc37a2*, which has a VDR-binding site within an intronic enhancer in the SI that is absent in the colon, as well as *Ptges*, which has an VDR binding, intronic enhancer in colon that is lower in the SI (Fig. 5*C*).

## Discussion

It is well established that 1,25(OH)_2_D_3_ is a critical regulator of intestinal physiology that controls calcium absorption (16), enhances barrier function (17, 18), regulates colonic inflammation (19), and suppresses colon cancer development (20). Despite these diverse effects, the genomic mechanisms used by 1,25(OH)_2_D_3_ to regulate intestinal biology remain unknown. Previous studies have focused on intestinal effects of 1,25(OH)_2_D_3_ in the mature small intestinal enterocyte related primarily to calcium absorption or protection against barrier dysfunction (17, 21). In contrast, effects of 1,25(OH)_2_D_3_ in small intestinal crypts are only beginning to be defined and have been a matter of debate (10, 13, 22). For example, it had been suggested that 1,25(OH)_2_D_3_ mediated transcription in intestinal villi but not in the crypts (23). We attempted to resolve this issue by examining the molecular actions of 1,25(OH)_2_D_3_ across multiple functional compartments in the intestine, that is, the SI crypt, the SI villus, and the colonic epithelium. Consistent with our recent study (8), 1,25(OH)_2_D_3_ treatment induced genes controlling intestinal Ca absorption in the all three compartments (*i.e.*, *Cyp24a1, Trpv6, S100g,* and *Atp2b1*). However, only a small number of the 1,25(OH)_2_D_3_-regulated gene targets were common across the three compartments (5.7% of the total DEG). Instead, the majority of 1,25(OH)_2_D_3_-mediated genomic events were distinct and compartment specific (Fig. 2). Thus, our study reveals a complexity to intestinal 1,25(OH)_2_D_3_ action that had not previously been appreciated in genomic studies using SI mucosal scrapings (14) or cultured cells (24, 25).

A significant amount of research has been conducted to define the mechanisms controlling intestine-specific and intestine-segment–specific gene expression. This has defined transcription factors like CDX2, HNF4a/g, and GATA4/5/6 as central regulators of intestine cell identity (26) and GATA6, SATB2, and KLF4 as colon-enriched transcription factors (15). However, few studies have explored how inducible gene expression is different across intestinal compartments. While there were only minor differences between VDR mRNA expression across the intestinal compartments, we found that ∼30% of compartment-specific 1,25(OH)_2_D_3_-induced transcripts could be explained by differential VDR binding to gene regulatory regions. Compartment-enriched VDR peaks were also found to differ in their enrichment of secondary transcription factor–binding motifs. This suggests that coordination between other transcription factors and VDR may contribute to differential binding and/or chromatin accessibility. For example, our findings of CDX2 and FOXA1 motifs at or near colon-enriched VDR-binding sites and HNF4a and GATA4 motifs at SI-enriched VDR-binding sites suggest that these transcription factors may promote or stabilize VDR binding at specific sites to mediate compartment-specific gene regulation. A stabilizing role would be similar to the role proposed for ETS1 in the regulation of 1,25(OH)_2_D_3_-mediated *Cyp24a1* gene expression (27). However, these hypotheses must be formally tested.

About 30% of the genes induced by 1,25(OH)_2_D_3_ in SI villus and colon and 48% of 1,25(OH)_2_D_3_-induced crypt transcripts did not have VDR-binding peaks. Also, between 60 to 79% of 1,25(OH)_2_D_3_-suppressed transcripts in each of the three compartments lacked a VDR-binding site. Thus, VDR binding does not predict mRNA expression. One hypothesis to explain VDR-independent and compartment-specific regulation of genes is that it is indirectly mediated through other transcription factors. In support of this hypothesis, we found 10 transcription factor genes that contained VDR-binding sites were differentially regulated by 1,25(OH)_2_ D treatment, and whose downstream targets were differentially regulated by 1,25(OH)_2_ D despite lacking VDR-binding sites near their genes. In the SI, this includes genes for transcription factors like NFATC3, which regulates intestinal differentiation (28), GATA4, which is a central regulator of proximal SI identity (26, 29, 30), and PDX1, which regulates enterocyte differentiation into enteroendocrine cells (31). Thus, 1,25(OH)_2_D_3_-mediated effects through these transcription factor genes could contribute to the prodifferentiating effects of the hormone in the intestine. In the colon, the mRNAs for *Hif1a* and *Klf5* were downregulated by 1,25(OH)_2_D and their genes included VDR-binding sites bound by VDR. In addition, we found that our list of 1,25(OH)_2_D_3_-regulated genes included suppression of HIF1A (*e.g.*, *Ncoa1, Mknk2, Tcf3*) and KLF5 target genes. KLF5 has been reported to regulate intestinal epithelial cell proliferation (32), particularly in the context of colon cancer (33). HIF1A has been reported to accelerate inflammatory responses (34) and 1,25(OH)_2_D_3_ signaling has recently been proposed by others to inhibit colitis by inhibiting HIF1A activation in colonic epithelial cells (35).

Lee *et al*. (14) previously examined the impact of 1,25(OH)_2_D treatment (10 ng/g BW, 6 h) on small intestinal gene expression in CYP27B1 KO mice using RNA-seq. Our work confirms some of their findings. For example, of the 72 transcripts we identified as 1,25(OH)_2_D_3_-regulated in all three compartments at 10% FDR (Fig. S2), more than 50% were previously were reported by Lee *et al*. This includes the classic intestinal 1,25(OH)_2_D_3_ target genes (*S100 g, Cyp24, Trpv6, Atp2b1*), the manganese exporter *Slc30a10* that we (8) and others (14, 36) have previously studied, and other genes whose functions are unrelated to mineral metabolism, that is, *Ppard*, *Shroom1, Bach1, Dhcr24*. BACH1 is a transcription factor that represses heme oxygenase gene transcription, inhibits NFE2L2 oxidative stress pathways, and is involved in the response to intestinal injury (37). DHCR24 is a dehydrocholesterol reductase involved in cholesterol biosynthesis (38) that can also exert antiapoptotic effects as a reactive oxygen scavenger (39). SHROOM1 is a member of the Shroom family of actin-binding proteins which have been reported to regulate cellular architecture in multiple tissues, including intestine (40). PPAR δ is a nuclear receptor that controls energy metabolism and cell survival (41) and may enhance intestinal stem cell function (42). While these proteins have not been extensively studied in the intestine, the fact that their genes all have VDR-binding sites, and their intestinal regulation by 1,25(OH)_2_D_3_ has been independently verified, suggest they are important mediators of 1,25(OH)_2_D_3_ function in the intestine.

Our work extends the findings of Lee *et al*. (14) by demonstrating that a number of 1,25(OH)_2_D_3_-induced genes were specific to one intestinal compartment. One such gene is *Ptges* that encodes prostaglandin E synthase, the terminal enzyme of the cyclooxygenase-mediated prostaglandin E2 (PGE2) biosynthesis pathway. This gene was found to be induced by 1,25(OH)_2_D_3_ and bound by VDR only in the colon. PGE2 is a bioactive lipid with diverse physiological roles including regulation of inflammatory responses (43). Although PGE2 has been reported to modulate gastrointestinal inflammatory responses, it has also been reported to have an important role in gut homeostasis by enhancing barrier function (44). A role for 1,25(OH)_2_D_3_ in protection against barrier dysfunction to inhibit colitis through regulation of proteins involved in cell-cell adhesion has previously been suggested (45, 46). Induction of *Ptges* by 1,25(OH)_2_D_3_ may be another mechanism whereby 1,25(OH)_2_D_3_ protects mucosal barrier function in the colon.

Among the genes selectively induced by 1,25(OH)_2_D_3_ in the SI but not in the colon are genes encoding drug metabolizing enzymes. Although it was previously suggested that the liver is the major site of xenobiotic metabolism, recent reports show that the intestine also has a major role in xenobiotic detoxification (47, 48). The drug metabolizing enzymes include phase I enzymes involved in enzymatic oxidation (*e.g.* cytochrome P450s) and phase II enzymes that catalyze conjugation reactions (*e.g.*, GST enzymes) (49). Phase I enzymes of the CYP1, 2, and 3 families contribute to the metabolism of the majority of xenobiotics (49). Among the CYPs, CYP3A4 contributes to the clearance of the greatest number of therapeutic agents and is also involved in 1,25(OH)_2_D_3_ catabolism (50, 51). Previous studies in humans and rats reported that 1,25(OH)_2_D_3_ regulates the expression of biotransformation enzyme genes in the intestine including *Cyp3a4*, UDP-glucuronosyltransferase, and GST Pi 1/2 class (52, 53). In our mouse study, we found SI-specific, 1,25(OH)_2_D_3_ induction of *Cyp3a11* (the mouse homolog of human *CYP3A4*), *Cyp2b10* (the human homolog is *CYP2B6*), *Gstm1,* and *Gstm3* mRNAs. Genes involved in xenobiotic metabolism are also induced by the xenobiotic-activated nuclear receptors PXR (pregnane X receptor) and Car (constitutive androstane receptor) (49). Similar to our study, the CAR ligand TCPOBOP induced *Cyp3a11*, *Cyp2b10,* and *Gmst1* and 3 mRNAs in mouse SI but not in colon, which has very low *Car* expression levels (54). In human intestine, expression of metabolizing enzyme genes like *Cyp2b6* and *Cyp3a4* was significantly higher in the SI than colonic tissue and was correlated to expression of PXR and CAR (48). The high induction of these genes in the SI may be a reflection that this is the site for most drug absorption (48). Regardless, additional studies are needed to determine whether segment-specific utilization of coregulatory factors contributes to this segment-specific regulation of xenobiotic metabolism by CAR, PXR, and VDR. In addition, future studies will be needed to determine if 1,25(OH)_2_D_3_ signaling can enhance intestinal drug or xenobiotic metabolism.

In addition to regulating genes for intestinal Ca absorption, 1,25(OH)_2_D_3_ treatment also regulated 45 solute transporter genes. This includes genes encoding transporters for various amino acids (*e.g.*, neutral amino acids, *Slc1a4, Slc43a2*), ion transporters (*e.g.*, K-Cl cotransporters, *Slc12a6, Slc12a7*), and organic molecules (*e.g.*, monocarboxylic acid, *Slc16a9, Slc16a13*). Similar to other reports (14), we also identified *Slc37a2* as strongly 1,25(OH)_2_D-induced in the SI and with VDR-binding sites associated with the gene. This is a glucose-phosphate transporter (55) whose expression in hematopoietic cells has been proposed as a biomarker for vitamin D status (56). However, the function of SC37A2 in the SI is unknown. Eight different zinc transporters were identified as 1,25(OH)_2_D_3_-regulated, including *Slc39a8* (encoding ZIP8) which was induced in all three segments and has VDR-binding sites in its gene. While gene variants of the human *SLC39A8* gene have been associated with Crohn’s Disease (57) its function in intestinal epithelial cells is not clear and requires additional study. We also identified three zinc transporters involved in intestinal zinc absorption as 1,25(OH)_2_D_3_-regulated; *Slc39a4* (encoding ZIP4) and *Slc30a5* (encoding ZNT5B), that mediate zinc uptake into cells, and *Scl30a1* (encoding ZNT1) that mediates zinc export at the basolateral membrane of enterocytes (58). These transporters are known to be regulated by zinc status (58, 59) and they were on the list of 1,25(OH)_2_D_3_-regulated genes in the intestinal RNA-seq study conducted by Lee *et al*. (14). We previously reported that 1,25(OH)_2_D_3_ treatment induces transcellular zinc transport across the human intestinal cell line, Caco-2 (60) but the mechanism for this effect was not determined. Further studies are needed to determine whether 1,25(OH)_2_D_3_ regulation of the *Slc39a4, Slc30a5, or Scl30a1* genes is important for intestinal zinc absorption.

The small intestinal crypt is an interesting compartment for 1,25(OH)_2_D_3_ action because it contains stem cells, proliferating daughter cells, and nonproliferating but undifferentiated cells. Expression of VDR and 1,25(OH)_2_D_3_ regulation of target genes in undifferentiated cells typically seen in crypts has been noted using human duodenal enteroids and human colon organoids (8, 61). Here, we found that crypt *Msi1* mRNA was induced by 1,25(OH)_2_D_3_, and DNA near the gene was bound by VDR. The *Msi1* gene encodes the RNA-binding protein Musushi1, a protein marker of intestinal stem cells and early intestinal cells lineages that is important in crypt regeneration (62). As such, this suggests a role for 1,25(OH)_2_D_3_ in intestinal stem cell renewal and the response to intestinal injury. Consistent with our observation in mice, RNA-seq analysis has shown that *MSI1* is also induced by 1,25(OH)_2_D_3_ in patient-derived colon stem cells (61). Other research showed that Lgr5+ stem cell–specific inactivation of VDR in mouse disrupted Lgr5+ stem cell function in mice (13). These findings suggest that 1,25(OH)_2_D_3_ has an important regulatory role not only in mature enterocytes but also in intestinal stem cells.

The strength of this study is that it is the most comprehensive examination of 1,25(OH)_2_D_3_-mediated intestinal gene regulation to date. The novelty of this study is that it is the first to define how 1,25(OH)_2_D_3_ action is influenced across critical functional axes within the intestine (*i.e.*, proximal/distal, SI crypt/villus). As a result, in addition to confirming many 1,25(OH)_2_D_3_-regulated target genes from earlier reports (14), our findings significantly expand our understanding of potential mechanisms by which 1,25(OH)_2_D_3_ alters the biology of various intestinal compartments. Still, we recognize this study has some limitations. First, we did not evaluate 1,25(OH)_2_D_3_ action on specific enterocyte lineages (*e.g.*, absorptive epithelial cells *versus* secretory cells like goblet cells, Paneth cells, and enteroendocrine cells) nor did we examine any potential age–related effects on intestinal 1,25(OH)_2_D_3_ action that might reflect age-associated intestinal resistance to the hormone (63, 64). Additionally, we used only one time for tissue harvest so we may have missed compartment-specific or gene-specific differences in 1,25(OH)_2_D_3_ responsiveness, for example, similar to the induction of *S100 g* (calbindin D_9k_) by 1,25(OH)_2_D_3_ that others reported was present in small intestinal villus but not in crypts (11). There is also a possibility that different cell extraction procedures required to isolate crypt epithelium *versus* duodenal epithelial compartments could lead to differences in measuring VDR binding between compartments—such potential differences should be considered when interpreting the data. Finally, additional studies are needed to validate the hypotheses we generated for new 1,25(OH)_2_D_3_ target genes and to test whether VDR-dependent recruitment of chromatin remodelers influences compartment-specific differences in chromatin accessibility and gene expression.

In summary, this study has expanded our understanding of how 1,25(OH)_2_D_3_ genomic action in different regions of the intestine may account for the compartment-specific, multiple regulatory actions of 1,25(OH)_2_D_3_ in the intestine. Further studies related to compartment-specific physiological functions of 1,25(OH)_2_D_3_, as well as a more comprehensive understanding of transcription factor networks involved in VDR mediated transcription, will provide new avenues of investigation related to the actions of 1,25(OH)_2_D_3_ in the regulation of intestinal physiology.

## Experimental procedures

### Mice and experimental design

All experiments were approved by the Animal Care and Use Committee at Rutgers University and at Rutgers, New Jersey Medical School. Mice were exposed to a 12h-light, 12h-dark cycle while food and water were given ad libitum.

#### RNA-seq experiment

C57BL/6J mice were obtained from The Jackson Laboratory. To maximize the transcriptional response to 1,25(OH)_2_ D, female mice were fed a vitamin D–deficient diet (Teklad, TD 89123, 0.4% Ca, 0.3% P, Envigo) for 2 to 3 weeks prior to mating, during pregnancy, and during lactation, and pups from the vitamin D–deficient dams were fed the vitamin D–deficient diet until 12 weeks of age. At the end of the experiment, mice (n=6–8 per group, balanced for sex) were injected ip with either 1,25(OH)_2_D_3_ (10 ng/g BW; Caymon Chemical Company) or vehicle (9:1 mix of propylene glycol:ethanol) and killed 4 h later. The time, dose of 1,25(OH)_2_D_3_, and use of vitamin D–deficient pups were chosen to maximize responsiveness of, and ability to detect, 1,25(OH)_2_D_3_-regulated transcripts. Ten to fifteen centimeter of the proximal SI was used to isolate crypts and villi while the entire colon was used for a mucosal scraping (specific sample preparation provided below).

#### VDR ChIP-Seq experiment

C57BL/6J mice were obtained from either the Jackson Laboratory or from breeding colonies maintained at Rutgers University. Mice were fed a standard rodent chow diet (Rodent Laboratory Chow 5001, Ralston Purina Co). At 10 to 12 weeks old, mice (n = 4 per treatment, balanced for sex) were treated ip with 1,25(OH)_2_D_3_ (10 ng/g body weight) and killed 1 h later. The dose and timing of 1,25(OH)_2_D_3_ treatment was chosen based on pilot ChIP-PCR studies that showed this treatment protocol enhanced VDR-binding peaks previously reported within enhancers of the *Cyp24a1* and *Trpv6* genes. Ten to fifteen centimeter of the proximal SI was used to isolate crypts and villi while the entire colon was used for a mucosal scraping (specific sample preparation provided below).

#### ATAC-seq experiment

C57BL/6J mice were obtained from either the Jackson Laboratory. Mice were fed a standard rodent chow diet (Rodent Laboratory Chow 5001, Ralston Purina Co) and small intestinal villus, small intestinal crypts, or colon was harvested at 12 weeks of age. We chose to use a normal chow diet and adult mice to capture the basal, physiologically relevant open chromatin regions in the mouse intestine. SI epithelium was separated into villus and crypt fractions, while the whole, unfractionated epithelium was used from colon (n = 3 per intestinal compartment).

### Crypt and villi isolation

The isolation of small intestinal crypts is a routine procedure in the area of intestinal biology (See Fig. S1 for typical results of an isolation). Preparations were isolated as we have described previously (65). For ChIP-seq, after isolation, the crypts and villi were incubated in 1.5% formaldehyde (Sigma-Aldrich, Cat. No. F8775-25 Ml), GibcoTM Advanced DMEM/F-12 (ThermoFisher Scientific, Cat. No. 12634010), and 1XPBS in rotator for 15 min at 4 °C and 40 min at 25 °C. After fixation, the samples were washed twice with 1XPBS for 3 min at 4 °C, then centrifuged at 200 rcf for 3 min at 4 °C and at 300 rcf for 30 s at 4 °C in order to remove any residual PBS. The samples were frozen on dry ice for 5 min before storage in the −80 °C freezer. For ATAC-seq, samples were prepared as we have previously described (66). For RNA-seq, Trizol was quickly added and the villus or crypt pellet (20–50 μl pellet per sample) was dispersed by pipetting, the samples were flash frozen in liquid nitrogen, and the samples were stored at −80 ^°^C.

### Colon whole epithelium isolation

The colon was harvested from the same group of C57BL/6J mice as the crypts and villi isolation at the same time. The entire colon from the terminal cecum to rectum were used for colon samples. After flushing with cold 1XPBS, the colon was opened longitudinally and the epithelial mucosa was removed by scraping until the colon became transparent. For ChIP-seq, the colon epithelial scraping was washed with 1XPBS twice and incubated in 1.5% formaldehyde solution in a rotator for 15 min at 4 °C and 40 min at 25 °C. After fixation, the sample was washed twice with 1XPBS for 3 min at 4 °C and spun down at 300 rcf for 30 s at 4 °C in order to remove any residual PBS. Finally, the samples were frozen on dry ice for 5 min before storage in the −80 °C freezer. For ATAC-seq, samples were prepared as we have previously described (66). For RNA-seq, Trizol was quickly added and the villus or crypt pellet was dispersed by pipetting, the samples were flash frozen in liquid nitrogen, and the samples were stored at −80 ^°^C.

### ChIP sample analysis

Three hundred microgram total cell pellets were used per ChIP replicate. The villi, crypts, and colon whole epithelium pellets were thawed on ice and mixed with 3 to 4 times the volume of the lysis buffer (1% SDS, 2% 0.5 M EDTA pH 8.0, 5% 1M Tris pH 8.0, and 10% 100X Mammalian ProteaseArrest protease cocktail (G-Biosciences, Cat. No. 786-433)) dissolved in MilliQ water). Lysates were incubated at RT for 10 min, aliquoted at volumes between 300 to 400 μl, and sonicated in cold ultrasonication water bath for intervals of 10 min. After sonication, 5 μl of the lysate was mixed with 100 μl of the reverse cross-linking buffer (10% 1M NaHCO3, 1% SDS, and MilliQ water) and incubated either overnight in 65 °C or for 15 min at 95 °C to reverse crosslinking. The DNA was purified from the lysate using QIAquick PCR Purification Kit (50) (Qiagen, Cat. No. 28104) and run on a 2% agarose gel to ensure chromatin size was between 200 to 500 bp. The sonicated lysate was centrifuged (15,000 rpm, 10 min, 8 °C), and five μl of the resulting supernatant was used as input. For immunoprecipitation, mixture of 15 μl of InvitrogenTM DynabeadsTM Protein A (ThermoFisher Scientific, Cat. No. 10008D) and 15 μl of InvitrogenTM DynabeadsTM Protein G (ThermoFisher Scientific, Cat. No. 10009D) were blocked in 1 ml of blocking buffer (10% 10X BSA, 10% 10X PBS, and MilliQ water) in rotator for an hour at 4 °C. Two anti-VDR antibodies from Santa Cruz Biotechnology were used for immunoprecipitation (D-6, Cat. No. sc-13133; C-20, Cat. No. sc-1008). ChIP samples were incubated with four μg of D-6 and two μg of C-20 or two μg of D-6 and four μg of C-20. Sonicated lysate and the supernatant dilution buffer (1% of 100X ProteaseArrestTM for Mammalian [100X], 2% 1M Tris pH 8, 3% 5M NaCl, 0.4% 0.5 M EDTA, 5% of 20% Triton X, and MilliQ water) were mixed together at a ratio that would give a concentration of 0.22 to 0.24% SDS. Immunoprecipitation was performed overnight at 4 °C, washed with 1 ml of RIPA buffer (5% 1M Hepes pH 7.6, 0.2% 0.5 M EDTA, 7% of 10% Na deoxycholate, 10% of 10% NP40, 12.5% 4M LiCl, and MilliQ water) with rotation for five times of 5 min at 4 °C. The RIPA buffer was removed and the samples were washed with 1 ml of TE buffer (0.1 mM EDTA, 10 mM Tris). The samples were then mixed with 100 μl of reverse cross-linking buffer and incubated for 6 h to overnight at 65 °C. The DNA was purified using QIAquick PCR Purification Kit and quantified using InvitrogenTM Quant-iTTM PicoGreenTM dsDNA Reagent (ThermoFisher Scientific, Cat. No. P7581) standards. ChIP DNA was used to prepare ChIP-seq libraries using the Takara Bio USA ThruPLEX DNA-seq Kit (R400427/R400428/R40048), and fragment size was selected using Pippin Prep and sequenced on an Illumina NextSeq system (2 x 75-bp reads; paired end; ∼14–18 M reads per sample).

### ChIP-seq data analysis

Sequencing adapters were removed from the read FASTQ files using NGmerge^26^. Each adapter-trimmed read FASTQ file generated by NGMerge was assessed using FastQC^22^. Each corresponding pair of forward and reverse adapter-trimmed read FASTQ files was aligned to the mm9 mouse genome assembly using Bowtie2^23^. Each alignment SAM file generated by Bowtie2 was converted to an alignment BAM file using the SAMtools^27^ suite. A composite alignment BAM file was constructed for each tissue by merging alignment BAM files from samples of the same compartment using the merge utility in the SAMtools^27^ suite. An alignment track file was generated from each alignment BAM file (both single-replicate and composite alignment BAM files) using the bamCoverage (deepTools^24^). Peak VDR-binding regions were identified in each alignment BAM file using the callpeak utility in MACS^28^. The resulting peak set files were filtered against the ENCODE blacklist for the mm9 genome assembly^29^. Pairwise comparisons using DiffBind^19^ (which includes sample normalization) were conducted to identify peaks exhibiting differential VDR-binding affinities between the intestinal compartments. Each set of differentially bound peaks was filtered to only include peaks, which were assigned a confidence value less than 0.001 by DiffBind. Each set of differentially bound peaks was exported to a BED file. A representative sample peak set BED file was generated from each cell type’s composite peak set BED file by randomly selecting 20% of the peaks in each composite peak set for use in HOMER motif calling analysis to save computational time. Several iterations of subsampling were compared and found to not majorly impact the results.

Raw and processed data files have been deposited into GEO as entry GSE161038. (https://www.ncbi.nlm.nih.gov/geo/query/acc.cgi?acc=GSE161038, token= **klufacscdfurnyv**).

### RNA-seq sample analysis

Samples in Trizol were thawed on ice and RNA was isolated using the RNeasy Plus Universal Kit with RiboZol RNA extraction reagent (Amresco), according to manufacturer’s instructions. All nucleic acid extracts were treated with gDNA Eliminator Solution for 15 s at 37 °C in order to remove contaminating chromosomal DNA. The resulting RNA was analyzed for quantity and quality with a NanoDrop spectrophotometer ND-1000 (Isogen Life Science) *via* an Agilent 2100 bioanalyzer. Only samples with RIN scores > 6.5 were used for analysis. RNA-seq was performed by BGISEQ-500 sequencing to generate 20,000 paired-end, 100 bp reads (BGI).

### RNA-seq data analysis

Kallisto (v0.45.0)^31^ was utilized to quantify the transcript abundances of the RNA-Seq samples with a RefSeq mm9 transcriptome build index. The tximport (v1.8.0)^32^ package was run in R (version 3.6.2) to create gene-level count matrices for use with DESeq2 (v1.2.0)^33^ by importing quantification data obtained from Kallisto. DESeq2 was then used to generate FPKM values per kilobase of gene length per million mapped fragments in each tissue sample with comparison of vitamin D–deficient replicates with 3 x D treated replicates. Two kinds of data were outputted from DESeq2: (1) a results table for reporting mean of normalized counts for all samples and through comparison of the treated condition *versus* untreated condition, log2 fold change, standard error, Wald statistic, Wald test *p*-value, and Benjamini-Hochberg adjusted *p*-value and (2) an FPKM table output of fragment counts normalized per kilobase of feature length per million mapped fragments.

Raw and processed data files have been deposited into GEO as entry GSE133949. (https://www.ncbi.nlm.nih.gov/geo/query/acc.cgi?acc=GSE133949, token=**kvaduyksbvindeb**).

### ATAC-seq sample and data analysis

The samples were prepared and analyzed as we have previously described (66). The ATAC-seq data are available in GEO as entry GSE134579.

### Secondary bioinformatic analyses

Enriched motifs were identified within VDR ChIP-seq peaks using HOMER findMotifsGenome.pl^25^. Genes associated with enriched peaks were identified by using GREAT^39^, run using the “single nearest gene” method within 10 kb parameter. Enriched gene sets produced by GREAT analysis was examined using GSEA^40^ (v2.2.1) and compared with gene expression data obtained from an RNA-seq assay of various intestinal regions in juvenile and adult mice.

Functional analysis of differentially expressed gene lists was conducted with the MetaCore analysis tool (Clarivate). Gene lists from the various compartments with 1,25(OH)_2_D_3_-mediated differential expression at 10% FDR were used for analysis so that subanalyses for upregulated or downregulated transcripts would contain > 200 differentially expressed genes. Analyses were conducted for pathway, GO processes, and protein class enrichment as well as transcription factor interactome analysis using a 5% FDR cut-off for significance. A list of all genes determined to be “present” in the intestinal compartments was used as the background gene set for analyses. The top 50 pathways or processes from each analysis were downloaded and lists for the three compartments were integrated for interpretation.

## Data availability

Raw and processed data files for the genomics experiments have been deposited into GEO as GSE133949 (RNA-seq), GSE161038 (VDR ChiP-Seq), and GSE134579 (ATAC-seq). In addition, summarized lists of differentially expressed genes and tables of the functional analysis the genomics data are presented in the supplementary materials as tables.

## Supporting information

This article contains supporting information.

## Conflict of interest

The authors declare that they have no conflicts of interest with the contents of this article.

## References

[1] Christakos S. (2021). Vitamin D: a critical regulator of intestinal physiology. JBMR Plus.

[2] Pike J.W., Christakos S. (2017). Biology and mechanisms of action of the vitamin D hormone. Endocrinol. Metab. Clin. North Am..

[3] Lee S.M., Bishop K.A., Goellner J.J., O'Brien C.A., Pike J.W. (2014). Mouse and human BAC transgenes recapitulate tissue-specific expression of the vitamin D receptor in mice and rescue the VDR-null phenotype. Endocrinology.

[4] Cartwright J.A., Gow A.G., Milne E., Drummond D., Smith S., Handel I. (2018). Vitamin D receptor expression in dogs. J. Vet. Intern. Med..

[5] Van Cromphaut S.J., Dewerchin M., Hoenderop J.G., Stockmans I., Van Herck E., Kato S. (2001). Duodenal calcium absorption in vitamin D receptor-knockout mice: Functional and molecular aspects. Proc. Natl. Acad. Sci. U. S. A..

[6] Xue Y.B., Fleet J.C. (2009). Intestinal vitamin D receptor is required for normal calcium and bone metabolism in mice. Gastroenterology.

[7] Dhawan P., Veldurthy V., Yehia G., Hsaio C., Porta A., Kim K.I. (2017). Transgenic expression of the vitamin D receptor restricted to the ileum, cecum, and colon of vitamin D receptor knockout mice rescues vitamin D receptor-dependent rickets. Endocrinology.

[8] Li S., De La Cruz J., Hutchens S., Mukhopadhyay S., Criss Z.K., Aita R. (2020). Analysis of 1,25-dihydroxyvitamin D3 genomic action reveals calcium-regulating and calcium-independent effects in mouse intestine and human enteroids. Mol. Cell Biol..

[9] Jiang H., Horst R.L., Koszewski N.J., Goff J.P., Christakos S., Fleet J.C. (2020). Targeting 1,25(OH)2D-mediated calcium absorption machinery in proximal colon with calcitriol glycosides and glucuronides. J. Steroid Biochem. Mol. Biol..

[10] Walters J.R., Weiser M.M. (1987). Calcium transport by rat duodenal villus and crypt basolateral membranes. Am. J. Physiol..

[11] Wu J.C., Smith M.W., Lawson D.E. (1992). Time dependency of 1,25(OH)2D3 induction of calbindin mRNA and calbindin expression in chick enterocytes during their differentiation along the crypt-villus axis. Differentiation.

[12] DeLuca H.F., Franceschi R.T., Halloran B.P., Massaro E.R. (1982). Molecular events involved in 1,25-dihydroxyvitamin D3 stimulation of intestinal calcium transport. Fed. Proc..

[13] Peregrina K., Houston M., Daroqui C., Dhima E., Sellers R.S., Augenlicht L.H. (2015). Vitamin D is a determinant of mouse intestinal Lgr5 stem cell functions. Carcinogenesis.

[14] Lee S.M., Riley E.M., Meyer M.B., Benkusky N.A., Plum L.A., DeLuca H.F. (2015). 1,25-Dihydroxyvitamin D3 controls a cohort of vitamin D receptor target genes in the proximal intestine that is enriched for calcium-regulating components. J. Biol. Chem..

[15] Zhou Q., Liu M., Xia X., Gong T., Feng J., Liu W. (2017). A mouse tissue transcription factor atlas. Nat. Commun..

[16] Fleet J.C. (2017). The role of vitamin D in the endocrinology controlling calcium homeostasis. Mol. Cell Endocrinol..

[17] Fujita H., Sugimoto K., Inatomi S., Maeda T., Osanai M., Uchiyama Y. (2008). Tight junction proteins claudin-2 and -12 are critical for vitamin D-dependent Ca2+ absorption between enterocytes. Mol. Biol. Cell.

[18] Kong J., Zhang Z., Musch M.W., Ning G., Sun J., Hart J. (2008). Novel role of the vitamin D receptor in maintaining the integrity of the intestinal mucosal barrier. Am. J. Physiol. Gastrointest Liver Physiol..

[19] Wang F., Johnson R.L., DeSmet M.L., Snyder P.W., Fairfax K.C., Fleet J.C. (2017). Vitamin D receptor-dependent signaling protects mice from dextran sulfate sodium-induced colitis. Endocrinology.

[20] Ferrer-Mayorga G., Larriba M.J., Crespo P., Munoz A. (2019). Mechanisms of action of vitamin D in colon cancer. J. Steroid Biochem. Mol. Biol..

[21] Song Y., Peng X., Porta A., Takanaga H., Peng J.B., Hediger M.A. (2003). Calcium transporter 1 and epithelial calcium channel messenger ribonucleic acid are differentially regulated by 1,25 dihydroxyvitamin D3 in the intestine and kidney of mice. Endocrinology.

[22] Colston K.W., Mackay A.G., Finlayson C., Wu J.C., Maxwell J.D. (1994). Localisation of vitamin D receptor in normal human duodenum and in patients with coeliac disease. Gut.

[23] Reynolds C.J., Koszewski N.J., Horst R.L., Beitz D.C., Goff J.P. (2019). Localization of the 1,25-dihydroxyvitamin d-mediated response in the intestines of mice. J. Steroid Biochem. Mol. Biol..

[24] Wood R.J., Tchack L., Angelo G., Pratt R.E., Sonna L.A. (2004). DNA microarray analysis of vitamin D-induced gene expression in a human colon carcinoma cell line. Physiol. Genomics..

[25] Costales-Carrera A., Fernandez-Barral A., Bustamante-Madrid P., Dominguez O., Guerra-Pastrian L., Cantero R. (2020). Comparative study of organoids from patient-derived normal and tumor colon and rectal tissue. Cancers (Basel).

[26] Vierstra J., Rynes E., Sandstrom R., Zhang M., Canfield T., Hansen R.S. (2014). Mouse regulatory DNA landscapes reveal global principles of cis-regulatory evolution. Science.

[27] Dwivedi P.P., Omdahl J.L., Kola I., Hume D.K., May B.K. (2000). Regulation of rat cytochrome P450C24 (CYP24) gene expression - evidence for functional cooperation of Ras-activated Ets transcription factors with the vitamin D receptor in 1,25-dihydroxyvitamin D-3-mediated induction. J. Biol. Chem..

[28] Wang Q., Zhou Y., Weiss H.L., Chow C.W., Evers B.M. (2011). NFATc1 regulation of TRAIL expression in human intestinal cells. PLoS One.

[29] Beuling E., Baffour-Awuah N.Y., Stapleton K.A., Aronson B.E., Noah T.K., Shroyer N.F. (2011). GATA factors regulate proliferation, differentiation, and gene expression in small intestine of mature mice. Gastroenterology.

[30] San Roman A.K., Aronson B.E., Krasinski S.D., Shivdasani R.A., Verzi M.P. (2015). Transcription factors GATA4 and HNF4A control distinct aspects of intestinal homeostasis in conjunction with transcription factor CDX2. J. Biol. Chem..

[31] Yamada S., Kojima H., Fujimiya M., Nakamura T., Kashiwagi A., Kikkawa R. (2001). Differentiation of immature enterocytes into enteroendocrine cells by Pdx1 overexpression. Am. J. Physiol. Gastrointest.Liver Physiol..

[32] Nandan M.O., Ghaleb A.M., Bialkowska A.B., Yang V.W. (2015). Kruppel-like factor 5 is essential for proliferation and survival of mouse intestinal epithelial stem cells. Stem Cell Res..

[33] McConnell B.B., Klapproth J.M., Sasaki M., Nandan M.O., Yang V.W. (2008). Kruppel-like factor 5 mediates transmissible murine colonic hyperplasia caused by Citrobacter rodentium infection. Gastroenterology.

[34] Corcoran S.E., O'Neill L.A. (2016). HIF1alpha and metabolic reprogramming in inflammation. J. Clin. Invest..

[35] Xue G., Gao R., Liu Z., Xu N., Cao Y., Zhao B. (2021). Vitamin D/VDR signaling inhibits colitis by suppressing HIF-1alpha activation in colonic epithelial cells. Am. J. Physiol. Gastrointest. Liver Physiol..

[36] Claro da Silva T., Hiller C., Gai Z., Kullak-Ublick G.A. (2016). Vitamin D3 transactivates the zinc and manganese transporter SLC30A10 *via* the Vitamin D receptor. J. Steroid Biochem. Mol. Biol..

[37] Harusato A., Naito Y., Takagi T., Yamada S., Mizushima K., Hirai Y. (2009). Inhibition of Bach1 ameliorates indomethacin-induced intestinal injury in mice. J. Physiol. Pharmacol..

[38] Luu W., Hart-Smith G., Sharpe L.J., Brown A.J. (2015). The terminal enzymes of cholesterol synthesis, DHCR24 and DHCR7, interact physically and functionally. J. Lipid Res..

[39] Lu X., Li Y., Liu J., Cao X., Wang X., Wang D. (2012). The membrane topological analysis of 3beta-hydroxysteroid-Delta24 reductase (DHCR24) on endoplasmic reticulum. J. Mol. Endocrinol..

[40] Dye D.E., Karlen S., Rohrbach B., Staub O., Braathen L.R., Eidne K.A. (2009). hShroom1 links a membrane bound protein to the actin cytoskeleton. Cell Mol. Life Sci..

[41] Giordano Attianese G.M., Desvergne B. (2015). Integrative and systemic approaches for evaluating PPARbeta/delta (PPARD) function. Nucl. Recept Signal..

[42] Beyaz S., Mana M.D., Yilmaz O.H. (2021). High-fat diet activates a PPAR-delta program to enhance intestinal stem cell function. Cell Stem Cell.

[43] Kalinski P. (2012). Regulation of immune responses by prostaglandin E2. J. Immunol..

[44] Morteau O. (2000). Prostaglandins and inflammation: the cyclooxygenase controversy. Arch. Immunol. Ther. Exp. (Warsz).

[45] Zhao H., Zhang H., Wu H., Li H., Liu L., Guo J. (2012). Protective role of 1,25(OH)2 vitamin D3 in the mucosal injury and epithelial barrier disruption in DSS-induced acute colitis in mice. BMC Gastroenterol..

[46] Chatterjee I., Zhang Y., Zhang J., Lu R., Xia Y., Sun J. (2021). Overexpression of vitamin D receptor in intestinal epithelia protects against colitis *via* upregulating tight junction protein claudin 15. J. Crohns Colitis.

[47] Lin J.H., Chiba M., Baillie T.A. (1999). Is the role of the small intestine in first-pass metabolism overemphasized?. Pharmacol. Rev..

[48] Fritz A., Busch D., Lapczuk J., Ostrowski M., Drozdzik M., Oswald S. (2019). Expression of clinically relevant drug-metabolizing enzymes along the human intestine and their correlation to drug transporters and nuclear receptors: an intra-subject analysis. Basic Clin. Pharmacol. Toxicol..

[49] Prakash C., Zuniga B., Song C.S., Jiang S., Cropper J., Park S. (2015). Nuclear receptors in drug metabolism, drug response and drug interactions. Nucl. Receptor Res..

[50] Guengerich F.P., McCarty K.D., Chapman J.G. (2021). Kinetics of cytochrome P450 3A4 inhibition by heterocyclic drugs defines a general sequential multistep binding process. J. Biol. Chem..

[51] Zhou C., Assem M., Tay J.C., Watkins P.B., Blumberg B., Schuetz E.G. (2006). Steroid and xenobiotic receptor and vitamin D receptor crosstalk mediates CYP24 expression and drug-induced osteomalacia. J. Clin. Invest..

[52] Kutuzova G.D., DeLuca H.F. (2006). 1,25-Dihydroxyvitamin D(3) regulates genes responsible for detoxification in intestine. Toxicol. Appl. Pharmacol..

[53] Wang Z., Lin Y.S., Zheng X.E., Senn T., Hashizume T., Scian M. (2012). An inducible cytochrome P450 3A4-dependent vitamin D catabolic pathway. Mol. Pharmacol..

[54] Park S., Cheng S.L., Cui J.Y. (2016). Characterizing drug-metabolizing enzymes and transporters that are bona fide CAR-target genes in mouse intestine. Acta Pharm. Sin B.

[55] Pan C.J., Chen S.Y., Jun H.S., Lin S.R., Mansfield B.C., Chou J.Y. (2011). SLC37A1 and SLC37A2 are phosphate-linked, glucose-6-phosphate antiporters. PLoS One.

[56] Wilfinger J., Seuter S., Tuomainen T.P., Virtanen J.K., Voutilainen S., Nurmi T. (2014). Primary vitamin D receptor target genes as biomarkers for the vitamin D3 status in the hematopoietic system. J. Nutr. Biochem..

[57] Li D., Achkar J.P., Haritunians T., Jacobs J.P., Hui K.Y., D'Amato M. (2016). A pleiotropic missense variant in SLC39A8 is associated with crohn's disease and human gut microbiome composition. Gastroenterology.

[58] Ohashi W., Hara T., Takagishi T., Hase K., Fukada T. (2019). Maintenance of intestinal epithelial homeostasis by zinc transporters. Dig. Dis. Sci..

[59] Hennigar S.R., Olson C.I., Kelley A.M., McClung J.P. (2021). Slc39a4 in the small intestine predicts zinc absorption and utilization: a comprehensive analysis of zinc transporter expression in response to diets of varied zinc content in young mice. J. Nutr. Biochem..

[60] Fleet J.C., Turnbull A.J., Bourcier M., Wood R.J. (1993). Vitamin D-sensitive and quinacrine-sensitive zinc transport in human intestinal cell line Caco-2. Am. J. Physiol..

[61] Fernandez-Barral A., Costales-Carrera A., Buira S.P., Jung P., Ferrer-Mayorga G., Larriba M.J. (2020). Vitamin D differentially regulates colon stem cells in patient-derived normal and tumor organoids. FEBS J..

[62] Chatterji P., Rustgi A.K. (2018). RNA binding proteins in intestinal epithelial biology and colorectal cancer. Trends Mol. Med..

[63] Wood R.J., Fleet J.C., Cashman K., Bruns M.E., DeLuca H.F. (1998). Intestinal calcium absorption in the aged rat: evidence of intestinal resistance to 1,25(OH)2 vitamin D. Endocrinology.

[64] Pattanaungkul S., Riggs B.L., Yergey A.L., Vieira N.E., O'Fallon W.M., Khosla S. (2000). Relationship of intestinal calcium absorption to 1,25-dihydroxyvitamin D [1,25(OH)2D] levels in young *versus* elderly women: Evidence for age- related intestinal resistance to 1,25(OH)2D action. J. Clin. Endocrinol. Metab..

[65] Chen L., Toke N.H., Luo S., Vasoya R.P., Aita R., Parthasarathy A. (2019). HNF4 factors control chromatin accessibility and are redundantly required for maturation of the fetal intestine. Development.

[66] Fleet J.C., Aldea D., Chen L., Christakos S., Verzi M. (2022). Regulatory domains controlling high intestinal vitamin D receptor gene expression are conserved in mouse and human. J. Biol. Chem..

