# Supplementary material for: Genomic analysis of 1,25-dihydroxyvitamin D3 action in mouse intestine reveals compartment and segment-specific gene regulatory effects
Source: J Biol Chem. 2022 Jun 30;298(8):102213. doi: 10.1016/j.jbc.2022.102213 (PMC9358460; doi:10.1016/j.jbc.2022.102213)

**Supplemental Figure S1.** Representative mages demonstrating the purity of small intestinal villus and crypt preparations. Details of the isolation procedure are presented in the Methods Section. Pictures were taken with a 4x objective. Scale bar represents 200 μm.

1. Small Intestinal Villi (B) Small Intestinal Crypts


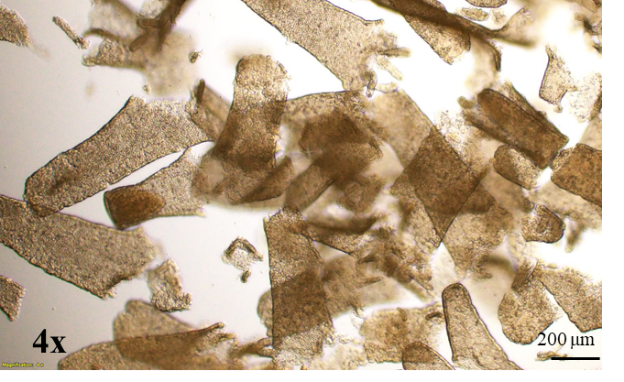

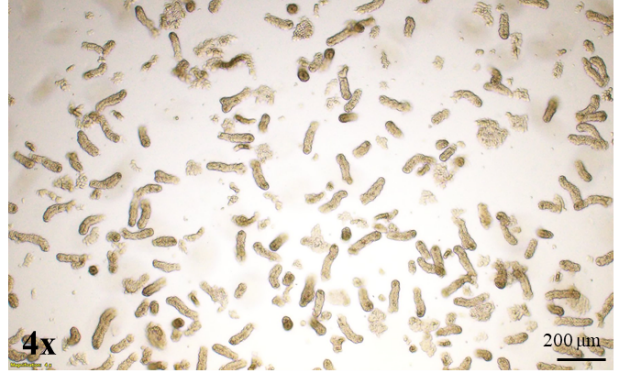


**Supplemental Figure S2.** Venn Diagram showing the vitamin D regulated genes across the three intestinal compartments (10% FDR)


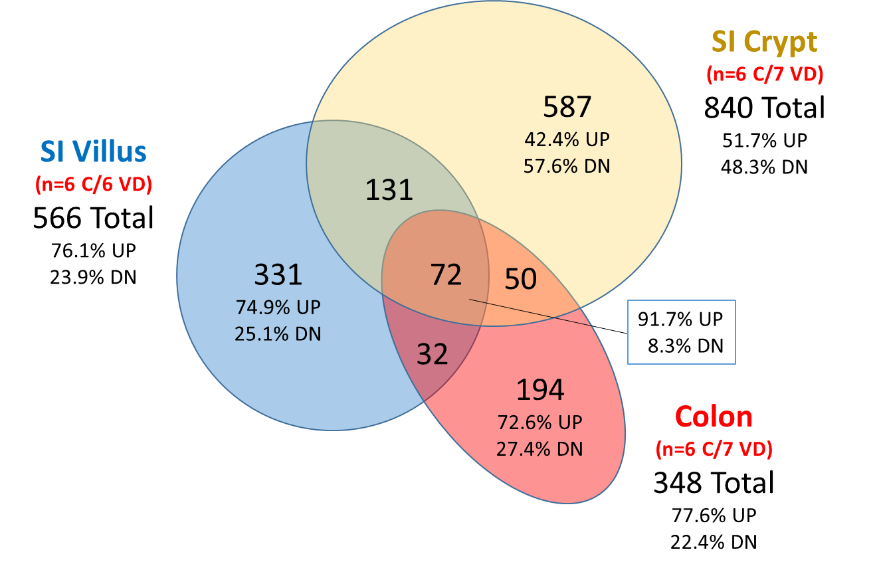


**Supplemental Figure S3.** Overlap between the vitamin D-regulated intestinal transcripts reported by Lee et al. (2016) J. Biol. Chem. 290:30573 and the 10% FDR list of vitamin D-regulated transcripts from the three intestinal compartments.


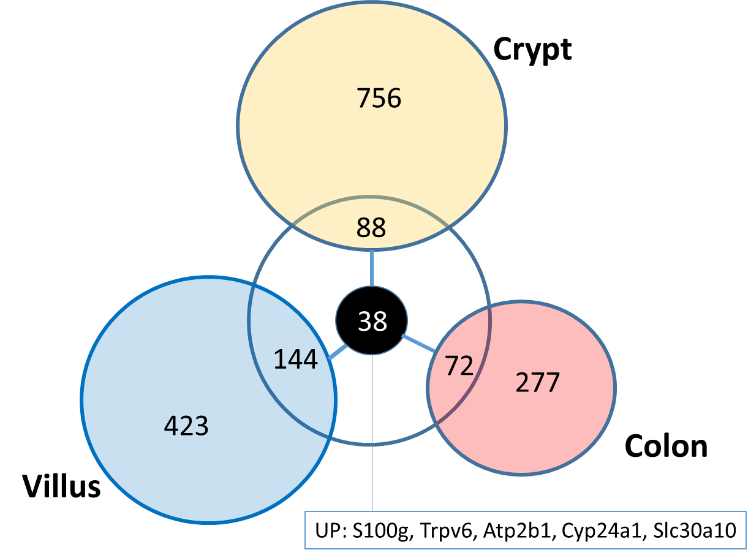


**Supplemental Figure S4.** VDR binding sites overlap with intestine, compartment ATAC-seq peaks demonstrating chromatin accessibility.

**
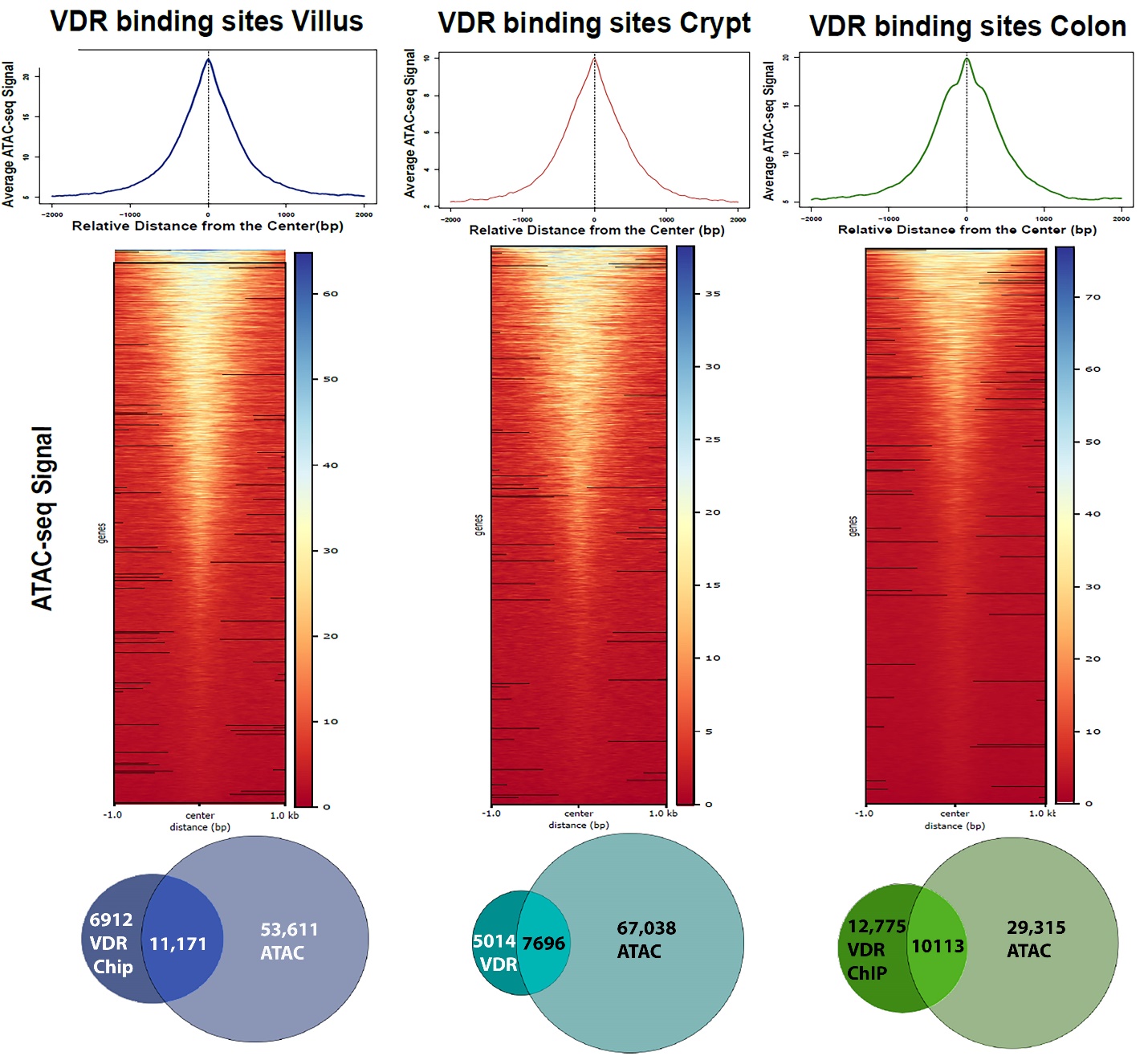
**

**Supplemental Figure S5.** Slc30a10 has VDR binding peaks that overlap with ATAC-seq peaks and others that do not.


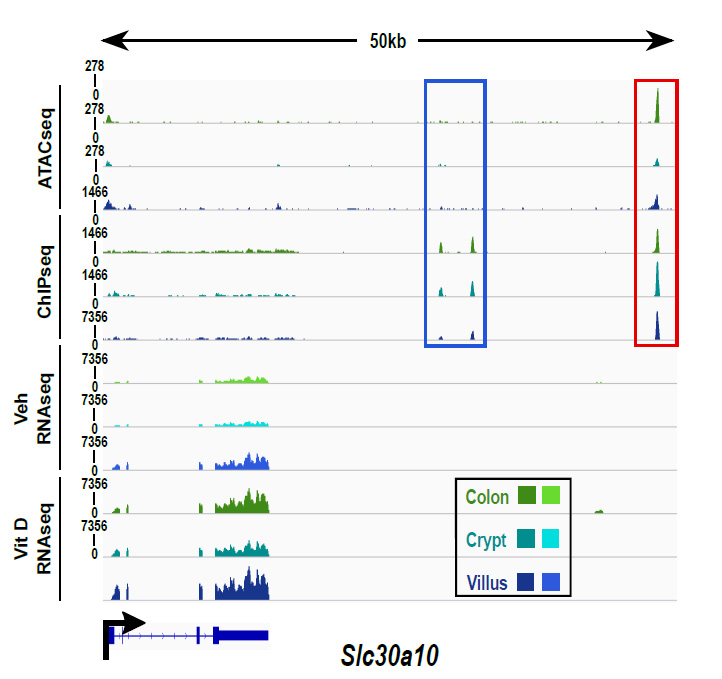


**Supplemental Figure S6.** Intestinal TF binding motifs that were enriched (A) across all VDR ChIP-seq peaks or (B) in VDR-ChIP-seq peaks with compartment-specific binding


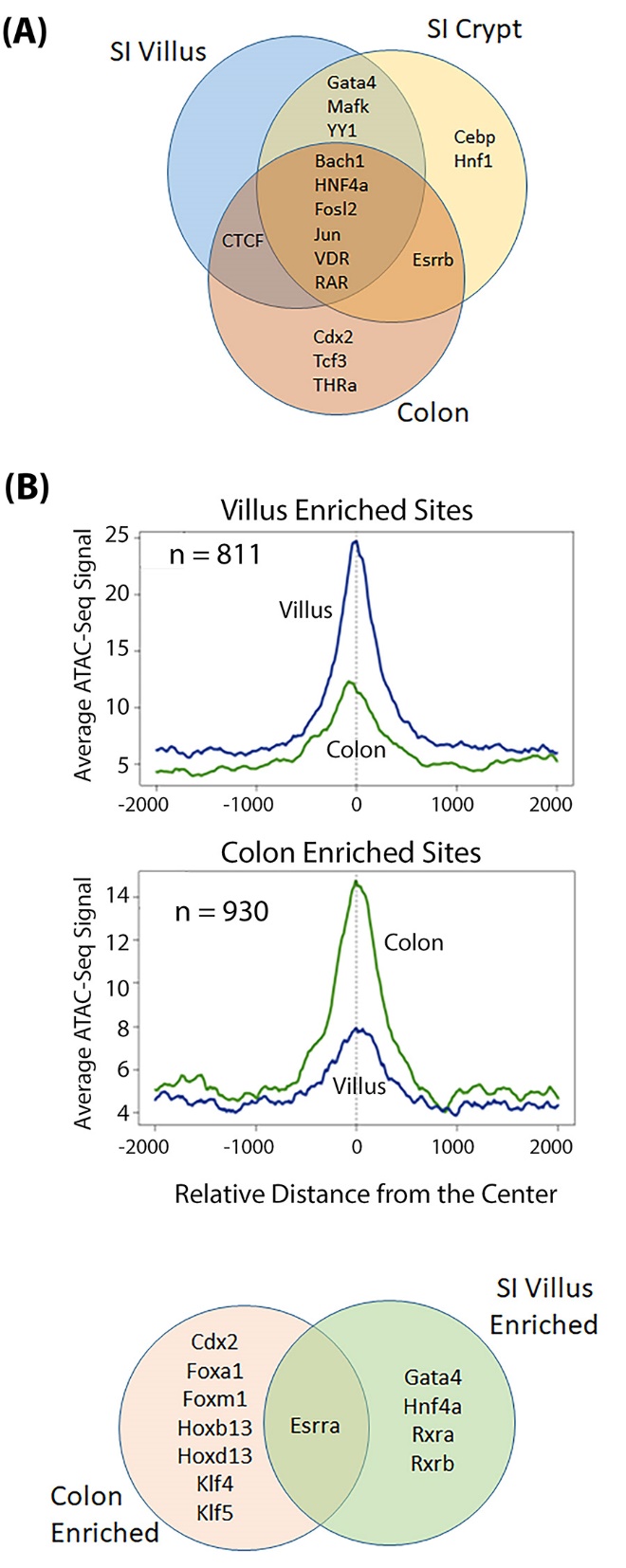

Supplement: Supplemental Figures S1–S6 [file mmc2.docx]
